# Hybridization Chain Reaction-Based Electrochemical Biosensors by Integrating the Advantages of Homogeneous Reaction and Heterogeneous Detection

**DOI:** 10.3390/bios13050543

**Published:** 2023-05-12

**Authors:** Ning Xia, Jiayou Cheng, Linxu Tian, Shuo Zhang, Yunqiu Wang, Gang Li

**Affiliations:** College of Chemistry and Chemical Engineering, Anyang Normal University, Anyang 455000, China

**Keywords:** hybridization chain reaction, electrochemical biosensors, homogeneous reaction, heterogeneous detection, glucose oxidase, streptavidin

## Abstract

The conventional hybridization chain reaction (HCR)-based electrochemical biosensors usually require the immobilization of probes on the electrode surface. This will limit the applications of biosensors due to the shortcomings of complex immobilization processes and low HCR efficiency. In this work, we proposed astrategy for the design of HCR-based electrochemical biosensors by integrating the advantages of homogeneous reaction and heterogeneous detection. Specifically, the targets triggered the autonomous cross-opening and hybridization oftwobiotin-labeled hairpin probes to form long-nicked dsDNA polymers. The HCR products with many biotin tags were then captured by a streptavidin-covered electrode, thus allowing for the attachment of streptavidin-conjugated signal reporters through streptavidin–biotin interactions. By employing DNA and microRNA-21 as the model targets and glucose oxidase as the signal reporter, the analytical performances of the HCR-based electrochemical biosensors were investigated. The detection limits of this method were found to be 0.6 fM and 1 fM for DNA and microRNA-21, respectively. The proposed strategy exhibited good reliability for target analysis in serum and cellular lysates. The strategy can be used to develop various HCR-based biosensors for a wide range of applications because sequence-specific oligonucleotides exhibit high binding affinity to a series of targets. In light of the high stability and commercial availability of streptavidin-modified materials, the strategy can be used for the design of different biosensors by changing the signal reporter and/or the sequence of hairpin probes.

## 1. Introduction

Hybridization chain reaction (HCR) refers to the process in which two hairpin DNA strands are alternately opened by a DNA initiator and then hybridized to form a long double-stranded DNA (dsDNA) with hundreds of repeated units [1]. One DNA initiator can induce a cascade of hybridization events, thus producing many nicked dsDNA hybrids and achieving signal amplification for target detection. As an enzyme-free and isothermal amplification technology, HCR has attracted much attention in practical applications due to its simple operation and mild reaction condition [2,3,4,5,6]. Since a few initiators can result in the formation of numerous hairpins, the HCR technique has been regarded as an alternative tool to other DNA-based signal-amplified methods including polymerase chain reaction (PCR), rolling circle amplification (RCA), catalytic hairpin assembly (CHA) and so on [7]. Differing from PCR, RCA, and CHA methods that require the use of nucleases and/or heating-cooling cycles for signal amplification, HCR is an enzyme-free technique that can be progressed isothermally. In recent years, HCR-based technologies have been widely used in the design of various optical and electrical biosensors, such as colorimetry, fluorescence, chemiluminescence, surface plasmon resonance (SPR), surface-enhanced Ramanspectra (SERS), electrochemistry, and inductively coupled plasma-mass spectrometry (ICP-MS) [3,8,9]. In comparison with other sensing devices, electrochemical biosensors exhibit the advantages of high sensitivity, low cost, excellent specificity, and easy miniaturization [10,11]. Moreover, the combination of HCR with other DNA amplification techniques, such as CHA, nuclease-assisted target recycling, and strand-displacement amplification (SDA), has further improved the detection sensitivity [12]. Although HCR-based strategies have exhibited good development momentum in the design of biosensors, most of the HCR-based electrochemical bioassays require the immobilization of DNA initiators on the electrode surface, which will suffer from some intrinsic shortcomings such as complex immobilization processes and low HCR efficiency due to the local steric hindrance [13,14,15]. Therefore, it is still necessary to develop an innovative, immobilization-free, and general strategy for the design of HCR-based electrochemical biosensors.

Homogeneous electrochemical biosensors can effectively overcome the problems of electrode modification, probe immobilization, and steric hindrance [16,17,18]. Such biosensors permit the molecular recognition and signal amplification reaction to be performed in a homogeneous solution with maximum reaction efficiency. For this view, many groups have been devoted to the development of homogeneous electrochemical sensing platforms [19,20,21,22,23,24,25,26,27]. A mostly classical strategy for the design of homogeneous electrochemical biosensors is based on the difference in the binding ability of reactants and products toward the sensing electrode [19,20,23,24,25]. For example, based on the difference in dispersion coefficient and adsorptive capacity of redox-labeled long-chain DNA and short nucleotides, various homogeneous electrochemical biosensors can be designed by target-initiated enzyme-assisted digestion reactions [23,24,25]. In addition, the intercalation of electroactive molecules or nanomaterials into the insitu formed G-quadruplex DNA or dsDNA and the stimuli-responsive release of encapsulated signal molecules from nanocarriers have also allowed for the development of homogeneous electrochemical sensing platforms [17,28]. In contrast to the homogeneous assay, heterogeneous detection shows the advantages of signal enhancement, less sample demand, and high sensitivity because the targets or signal molecules are concentrated on a limited solid surface [29,30,31,32]. Thus, in this work, we attempted to propose a general HCR-based strategy for the design of electrochemical biosensors by integrating the advantages of homogeneous reactions and heterogeneous detection.

**Scheme 1:**
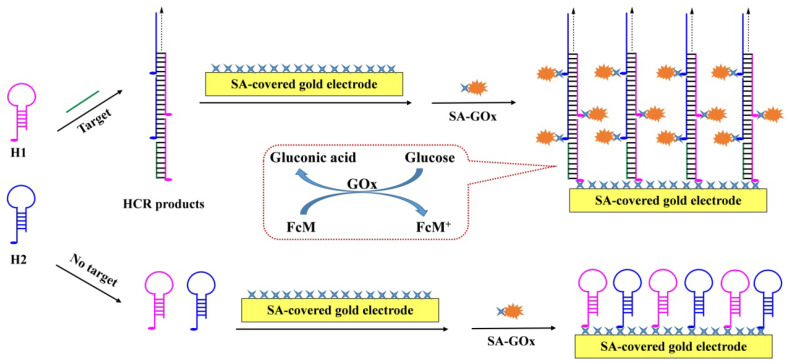
Schematic representation for the HCR-based electrochemical biosensor by integrating the advantages of homogeneous reaction and heterogeneous detection.

The general strategies for HCR-based electrochemical biosensors involve the label of hairpin DNA strands with signal tags such as catalytic elements (e.g., natural enzymes, DNAzymes, and nanocatalysts) and electroactive nanoparticles or small molecules [10,33,34]. Among them, the catalytic signal amplification can make the HCR-based assays highly sensitive to achieve the detection of low abundance targets. In addition, the interaction between streptavidin (SA) or avidin and biotin is the commonly used system for labeling biomolecules with catalytic elements including natural or mimetic enzymes and nanocatalysts [35,36,37]. In addition, SA-functionalized solid substrates have been extensively applied for the separation and immobilization of biotinylated biomolecules [30]. For example, SA-modified electrodes and chips have been widely used for the immobilization of biotinylated acceptors such as antibodies, peptides, and DNA. SA-conjugated nanomaterials and quantum dots have been used as signal reporters by binding to the biotinylated recognition elements. Herein, we suggested that SA-modified sensing electrodes could be used for the design of HCR-based electrochemical biosensors by integrating the advantages of homogeneous reaction and heterogeneous detection (Scheme 1). The two hairpin DNA strands were labeled with biotin tags for the attachment of catalytic elements through streptavidin–biotin interactions. When the HCR was triggered by the initiator in solution, the resulting biotin-dsDNA (H1/H2) hybrids were captured by the sensing electrode through SA–biotin interactions. Other biotin tags in the HCR products would induce the attachment of SA-conjugated catalytic elements such as glucose oxidase (GOx) to the sensing electrode. In the absence of the DNA initiator, the two biotinylated hairpin DNA strands were captured by the SA-modified electrode and no SA-GOx conjugates were attached to the electrode. In this method, the HCR was performed in solution and the products/signal molecules were gathered onto the electrode surface, thus integrating the advantages of homogeneous reaction and heterogeneous detection. In addition, SA proteins are extremely stable, and SA-modified electrodes can decrease nonspecific adsorption. The reports of HCR-based biosensors for the detection of nucleic acids, metabolites, proteins, toxins, andmetal ions are innumerable [7]. Therefore, through the rational design of DNA initiators and hairpin probes, we believe that the strategy could be used for the development of other HCR-based biosensors for the detection of a variety of analytes.

## 2. Materials and Methods

### 2.1. Chemicals and Reagents

SA, GOx, Trizol reagent, and oligonucleotides were provided by Sangon Biotech. Co., Ltd. (Shanghai, China). 6-Sulfanylhexanoic acid, ferrocenemethanol(FcM), 1-ethyl-3(3-1-ethyl-3(3-dimethylaminopropyl)) carbodiimide hydrochloride (EDC), N-hydroxysulfosuccinimide (NHS) and sulfo-NHS-biotin were ordered from Sigma-Aldrich (Shanghai, China). The blood was provided by the medical center of Anyang Normal University (Anyang, China). The cell lines were provided by the Institute of Surface Analysisand Biosensing at Central South University (Changsha, China). Other chemicals were of analytical grade and used without additional treatment. The solutions were prepared and diluted with purified water from a Milli-Q purification system.

The sequences of initiator DNA, miRNA-21, and hairpin probes used in this work were designed according to the previous reports [1,38]. Their sequences are shown as follows: AGT CTA GGA TTC GGC GTG GGT TAA (initiator DNA), AGT CTA GGA TTG GGC GTG GGT TAA (single-base mismatched), AGT CTT GGA TTG GGC GTG GGA TAA (three-base mismatched), CGT GTA GGT TAT GAC GTG CGT AGC (random), biotin-TTA ACC CAC GCC GAA TCC TAG ACT CAA AGT AGT CTA GGA TTC GGC GTG (H1), biotin-AGT CTA GGA TTC GGC GTG GGT TAA CAC GCC GAA TCC TAG ACT ACT TTG (H2), UAG CUU AUC AGA CUG AUG UUG A (miRNA-21), biotin-CAG ACT GAT GTT GAC GTG AAA CTC AAC ATC AGT CTG ATA AGC TA (H3), and biotin-GTT TCA CGT CAA CAT CAG TCT GTA GCT TAT CAG ACT GAT GTT GA (H4).

### 2.2. Preparation of SA-Covered Electrode

The gold disk electrode was treated with a mixture of 98% H_2_SO_4_ and 30% H_2_O_2_ at a volume ratio of 3:1 for 30 min. After being thoroughly rinsed with pure water, the electrode was polished with 0.05 μm alumina. Then, the electrode was cleaned with 50% ethanol under sonication for 1 min. After removing the residual alumina powders, the electrode was electrochemically cleaned in 0.5 M H_2_SO_4_ by the cyclic voltammetry method. The scanning potential changed in the range of −0.2 and 1.6 V and the scanning rate was 100mV/s. When a stable voltammetric curve was attained, the electrode was cleaned with pure water, dried with nitrogen, and then incubated with 6-sulfanylhexanoic acid solution overnight. After that, the electrode was incubated with a mixture of 0.2 mM EDC and 0.1 mM NHS for 15 min. This step was followed by incubating the electrode with 10 μL of 1 mg/mL SA in phosphate buffer (10 mM, pH 7.4) for 2 h. Then, the electrode was incubated with 1mM ethylamine for 30 min to block the unreacted activated carboxyl groups. After being washed thoroughly with water, the electrode was used for the capture of HCR products.

### 2.3. Preparation of SA-GOx Conjugates

Although nanotechnology has promoted the rapid development of nanocatalysts, enzymatic signal amplification is still the commonly used strategy for bioassays in practical applications, including horseradish peroxidase (HRP), GOx, and alkaline phosphatase (ALP). Enzyme or fluorophore-labeled SA conjugates are commercially available in a wide range of application domains. Herein, GOx was used as the signal reporter. The SA-GOx was prepared by conjugation of SA and biotinylated GOx (biotin-GOx) through avidin–biotin interaction [39]. Biotin-GOx was prepared by incubation of 100 μM sulfo-NHS-biotin with 0.5 mg/mL GOx in 1 mL of phosphate buffer for 2 h. The unreacted sulfo-NHS-biotin was removed by a centrifugal filtration device with a 10,000 cut-off. Then, 100 μL of SA at an optimized concentration of 2.5 mg/mL was mixed with the prepared biotin-GOx. The resulting SA-GOx conjugates were stored at 4°C for use.

### 2.4. Assays of DNA

The hairpin probes (H1 and H2) were individually dissolved in an RNase-free Tris solution (20 mM, pH 7.4) containing 0.1 M NaCl (TNE buffer). After being heated at 95 °C for 5 min, the probe solution was cooled slowly to room temperature. Then, 10 μL of DNA sample was incubated with 10 μL of the mixture of two hairpin probes at 37 °C for a given time. After that, the reaction solution with a volume of 5 μL was coated onto the SA-covered electrode surface. After reaction for 10 min, the electrode was rinsed with TNE buffer and then exposed to 3 μL of SA-GOx solution. After incubation for 10 min again, the electrode was thoroughly washed with water and then placed in the mixture of 100 μM FcM and 5 mM glucose for voltammetric measurement.

For analysis of DNA in real samples, the blood was centrifuged at 2000 rpm for 5 min and then 100 μL of serum was taken out and diluted with TNE buffer to 1 mL. Then, DNA at a given concentration was spiked into the serum for analysis. The other steps followed those for the analysis of DNA standard samples.

### 2.5. Detection of miRNA-21

The hairpin H3/H4 probes were first heated individually at 95 °C for 5 min and then cooled slowly to room temperature. For the assays of miRNA-21, the storage sample was diluted at different times with RNase-free TNE buffer. Then, 10 μL of miRNA-21 sample was incubated with 10 μL of H3/H4 mixture at 37 °C for 60 min. The SA-covered electrode was exposed to 5 μL of the reaction solution for 10 min, and then thoroughly rinsed with TNE buffer. After that, 3 μL of SA-GOx solution was coated onto the electrode to incubate for 10 min. After being thoroughly washed with water, the electrode was placed in the FcM/glucose mixture for voltammetric measurement.

### 2.6. Extraction of Total RNA from Cancer Cells

The cell lysates were extracted from cancer cells using the procedures described in our previous reports [30,32]. In brief, the MCF-7 and HeLa cancer cells were cultivated at 37 °C in Dulbecco’s modified Eagle’s medium (DMEM) containing 10% FBS and 5% penicillin-streptomycin with a humidified atmosphere (95% air and 5% CO_2_). After the cells were counted and washed, the Trizol reagent was addedto extract a total RNA sample according to the manufacturer’s protocol. The resulting cellular lysates were stored at −80 °C for use. Before the quantification of miRNA-21 in the lysates, the samples were diluted by different folds with the RNase-free TNE buffer. Then, 10 μL of sample was incubated with 10 μL of the H3/H4 mixture at 37 °C for 60 min. Other experimental steps were the same as those for the assays of miRNA-21 standard samples.

## 3. Results and Discussion

### 3.1. Detection Principle

The detection principle of the HCR-based electrochemical biosensor is depicted in Scheme 1. Two hairpin probes named H1 and H2 were labeled with biotin tags, respectively. The presence of target DNA could trigger the autonomous cross-opening of two hairpin probes to form long-nicked biotin-dsDNA polymers. The HCR products contain many biotin tags, which would favor the attachment of biotin-dsDNA polymers to the SA-covered electrode through avidin–biotin interactions. Other biotin tags in the polymers could capture SA-GOx conjugates through the same interactions. The attached GOx could electrochemically catalyze the oxidation of glucose with FcM as the redox mediator, thus causing a dramatic enhancement in the peak current. In the absence of initiator DNA, the hairpin probes were attached to the electrode surface by binding to the immobilized SA molecules. Since each hairpin probe contains one biotin tag, the SA-GOx conjugates could not be captured by the sensing electrode and there was no obvious increase in the peak current. The electrochemical signals would be proportional to the number of HCR products or the concentration of initiator DNA. The method may allow for target detection with high sensitivity due to the double signal amplification of HCR and GOx.

### 3.2. Feasibility of This Method

**Figure 1:**
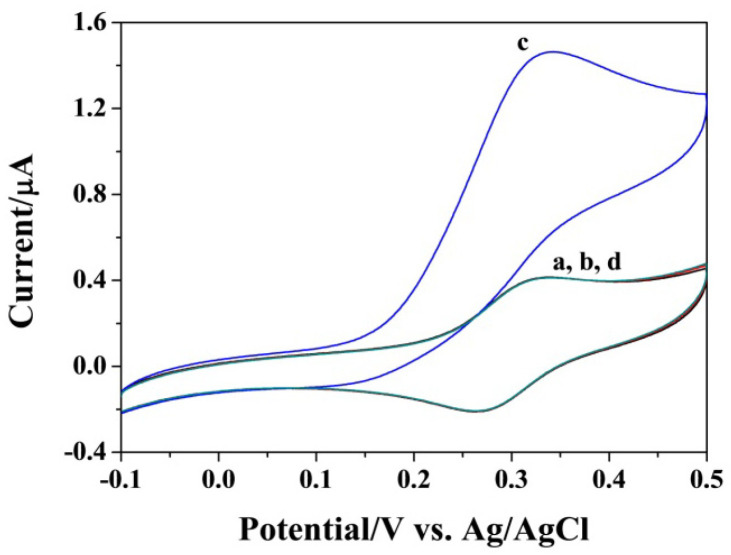
Representative cyclic voltammograms (CVs) of SA-covered gold electrode in the FcM/glucose solution with the incubation of initiator DNA (curve a), H1/H2 (curve b), initiator DNA/H1/H2 (curve c) and initiator DNA/H2 (curve d) before the incubation step of GOx. The concentrations of DNA and H1/H2 were 5 pM and 1 nM.

Figure 1 shows the results ofexploring the feasibility of the strategy. Without initiator DNA or hairpin probes (curve a and b), the redox peak of FcM was reversible even when the SA-covered electrode was incubated with SA-GOx, indicating that the SA-GOx conjugates could not be captured by the modified electrode. When the electrode was incubated with the mixture of initiator DNA and hairpin probes and then treated with SA-GOx solution (curve c), a significant increase in the anodic current was obtained. Another control experiment was conducted in the absence of the H2 probe (curve d). Consequently, no obvious increase in the peak current was observed, suggesting that the HCR process was dependent on two hairpin probes. These results also demonstrated that the system could be used to determine the target DNA by using an SA-modified electrode to capture the HCR products formed in a homogeneous solution.

**Figure 2:**
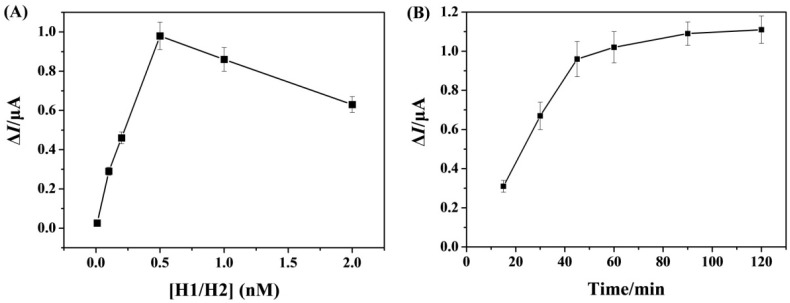
Dependence of Δ*I* on the concentration of hairpin H1/H2 probes (**A**) and the incubation time (**B**).

To achieve high sensitivity, the experimental parameters such as the concentration of hairpin probes and the incubation time for HCR were investigated. As shown in Figure 2A, a maximal change in anodic current (Δ*I*) was obtained when the concentration of hairpin H1/H2 probes was 0.5 nM. Beyond the value, the current began to decrease, probably due to the competitive reaction between the free hairpin probes and the HCR products to bind with SA on the electrode surface. We also found that the peak current increased with the increase in incubation time for the HCR process (Figure 2B). In light of operation convenience and detection sensitivity, 1 h was chosen as the optimized incubation time.

### 3.3. Analytical Performances for DNA Detection

**Figure 3:**
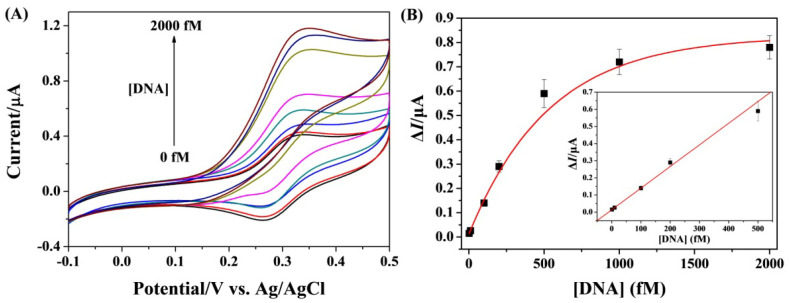
(**A**) CVs for the detection of various concentrations of DNA (from bottom to top: 0, 1, 10, 100, 200, 500, 1000, and 2000 fM). (**B**) Fitting curve of Δ*I* against DNA concentration. The inset depicts the linear portion of the curve.

The analytical performances of this method were first studied by measuring different concentrations of target DNA. As depicted in Figure 3A, the increase in DNA concentration caused the gradual increase in the anodic peak current, suggesting that a higher concentration of DNA could trigger the formation of more HCR products. From the relationship between peak current and DNA concentration, a good linear calibration curve was attained in the range of 1~500 fM (Figure 3B). The linear equation can be expressed as Δ*I* = 0.0137 + 0.0013[DNA] (fM). The detection limit was found to be 0.6 fM based on the 3*σ*/slope method. The value is comparable to or even lower than those of other electrochemical methods with the signal amplification of HCR plus nanomaterials or enzymatic isothermal reactions (Table 1). The high sensitivity can be attributed to the integration of the advantages of homogeneous reaction and heterogeneous detection.

**Figure 4:**
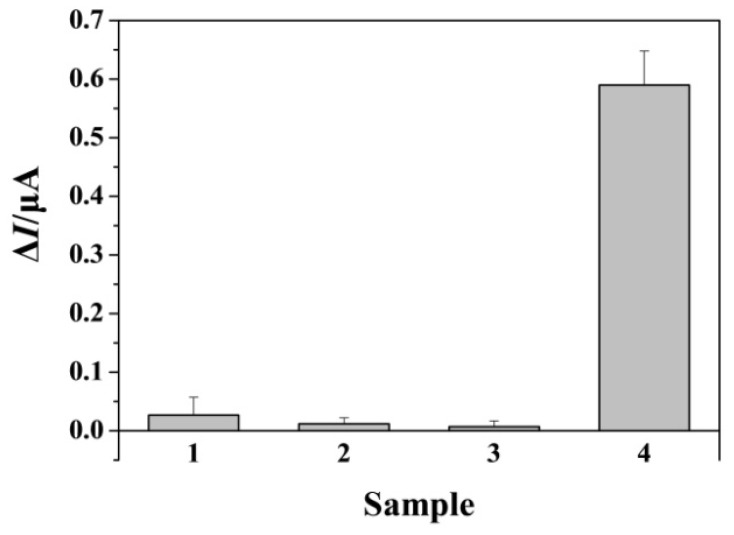
Selectivity of the method toward single-base mismatched (bar 1), three-base mismatched (bar 2), random (bar 3), and target (bar 4) DNA. The concentration of target DNA was 500 fM and that of other oligonucleotides was 10 pM.

To explore the specificity, the target and its single-base mismatched, three-base mismatched, and random sequences were analyzed. Figure 4 shows the change in the anodic peak currents for the assays of different DNA strands. It was found that the Δ*I* for target DNA was significantly higher than that for other oligonucleotides. This suggested that the method exhibited good specificity to discriminate the target from base mismatched sequences.

### 3.4. Assays of DNA in Serum Samples

**Figure 5:**
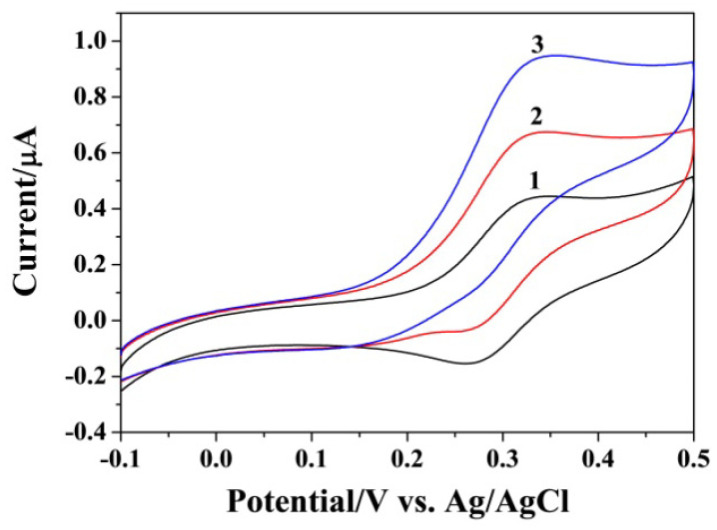
CVs for the assays of target DNA spiked in serum.

To verify the applicability, the biosensor was used to determine DNA in a serum sample. The serum was spiked with three concentrations of target DNA (20, 200, and 400 fM) and then analyzed. As shown in Figure 5, the anodic peak current was intensified with an increasing concentration of DNA. The recoveries of the assays ranged from 92% to 105% with the relative standard deviations below 12%. The result demonstrated that the biosensor exhibited good applicability in complex biological environments.

### 3.5. Quantification of miRNA-21

MiRNAs are a class of short non-coding single-stranded RNA stands. They play an important role in many physiological processes, including gene regulation, cell differentiation, cellproliferation, and apoptosis. Much evidence has suggested that miRNAs were intimately related to theprogression of different diseases (e.g., cancers, heartdiseases, and diabetes) [54,55]. Thus, miRNAs have been regarded as promising disease biomarkers. Quantification of their levels will be of great importance for early diagnosis and therapeutic assessment of many diseases. Although many HCR-based methods have been developed to determine miRNAs [56,57], they may suffer from the limitations of complex procedures and low sensitivity or involve the use of multiple signal amplification by nucleases and nanomaterials [13,14,15]. Considering the high sensitivity, selectivity, and simplicity of the proposed electrochemical method, miRNA-21 was quantified as an example analyte. The detection principle was similar to that for the DNA assay as shown in Scheme 1, where the hairpin probes of H1 and H2 were replaced by H3 and H4.

**Figure 6:**
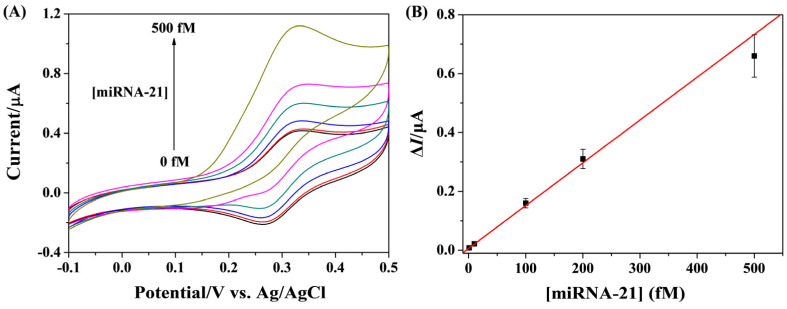
(**A**) CVs for the detection of various concentrations of miRNA-21 (from bottom to top: 0, 1, 10, 100, 200, and 500 fM). (**B**) Fitting curve of Δ*I* against miRNA-21 concentration.

The dynamic range of the method for miRNA-21 quantification was evaluated by measuring different concentrations of the target. As shown in Figure 6A, the anodic peak current was enhanced continuously with the increase in miRNA-21 concentration. A good linearity between the current change and miRNA-21 concentration was attained in the range of 1~500 fM (Figure 6B). The linear equation can be expressed as Δ*I* = 0.0068 + 0.0015[miRNA-21] (fM). The lowest detectable concentration is comparable with or even lower than those of other HCR-based methods (Table 1). In contrast to other signal amplification strategies such as HRP, ALP, nucleases, and nanomaterials (Table 2), our method, by integrating the advantages of homogeneous reaction and heterogeneous detection, exhibited high simplicity and sensitivity, posing a significant contribution to the sensitive detection of miRNAs.

### 3.6. Assays of miRNA-21 in Cellular Lysates

**Figure 7:**
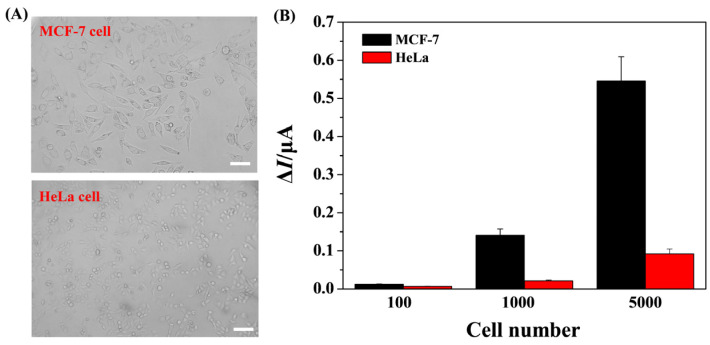
(**A**) Morphological images of MCF-7 and HeLa cells. The scale bar is 50 μm. (**B**) Results for the quantification of miRNA-21 in cellular lysates extracted from MCF-7 and HeLa cells.

To further evaluate the practicality of the biosensor, miRNA-21 in cellular lysates extracted from MCF-7 cells (breast cancer cell line) and HeLa cells (cervical cell line) (Figure 7A) were analyzed. As shown in Figure 7B, the anodic peak current was sharply intensified with increasing MCF-7 cell numbers. A smaller increasing trend between Δ*I* and cell number was found for the assays of miRNA-21 in HeLa cells. In addition, the anodic peak currents for MCF-7 cells were higher than those for HeLa cells. These results indicated that the level of miRNA-21 in MCF-7 cells is higher than that in HeLa cells, which is consistent with previous reports [30,32,38,67]. Thus, the proposed biosensor shows high practicality for the assays of biological samples, providing an alternate tool for clinical diagnosis and therapeutic assessment of diseases.

## 4. Conclusions

In summary, we proposed a simple and sensitive HCR-based sensing strategy by integrating the advantages of homogeneous reaction and heterogeneous detection. The biosensor was constructed with GOx as the signal label on the basis of avidin–biotin interaction. The homogeneous reaction improved the HCR efficiency and the heterogeneous detection endowed the biosensor with high sensitivity. The biotin tags in the HCR products allowed for the attachment of many signal labels to the electrode surface, thus leading to secondary signal amplification. This strategy can improve the sensing reliability and reduce the false positive signal caused by nonspecific adsorption. The proposed biosensor exhibited high sensitivity and reliability for the analysis of DNA and miRNA-21. The detection limits were found to be 0.6 fM and 1 fM for DNA and miRNA-21, respectively. Furthermore, the method was used to quantify miRNA-21 in cellular lysates derived from two cancer cells (MCF-7 and HeLa) and the result is accordant with that of the previous reports. The strategy can be used to develop various HCR-based sensing platforms for a wide range of applications because the oligonucleotides exhibit specific binding affinity to a series of targets.

## Figures and Tables

**Table 1:** Analytical performances of HCR-based electrochemical nucleic acid biosensors.

Signal Amplification	Target	Linear Range	Detection Limit	Ref.
HRP	DNA	2.5 fM	10 fM~0.1 nM	[40]
HRP	miRNA	10 aM	10 aM~1 pM	[41]
DNA^TT^ + HRP	miRNA	0.1 fM	0.1 fM~1 nM	[42]
ALP	miRNA	0.56 fM	1 fM~100 pM	[43]
CSDPR + ALP	DNA	8 fM	10 fM~1 nM	[44]
WCHA + ALP	DNA	8.3 fM	10 fM~5 nM	[45]
AuNPs + AgNPs	miRNA	2 aM	100 aM~100 pM	[46]
Fe_3_O_4_/Thi or Fe_3_O_4_/Fc	miRNA	0.44 fM	1 fM~1 nM	[47]
ASDA + AgNCs	DNA	0.16 fM	0.2 fM~1 pM	[48]
TAPNR + AgNCs	miRNA	0.64 fM	1.0 fM~0.1 nM	[49]
CuCo-CeO_2_	miRNA	0.05 fM	0.1 fM~10 nM	[50]
Exo-III/RuHex	DNA	0.2 fM	1 fM~1.0 nM	[51]
CHA + MB	DNA	0.037 fM	0.1 fM to 5.0 pM	[52]
Exo I/RuHex	miRNA	53 aM	0.1 fM to 0.1 μM	[53]
GOx	DNA	0.6 fM	1~500 fM	This work

Abbreviations: HRP, horseradish peroxidase; DNA^TT^, DNA tetrahedron; CSDPR, circular strand-displacement polymerase reaction; ALP, alkaline phosphatase; WCHA, catalytic hairpin assembly; AuNPs, gold nanoparticles; AgNPs, silver nanoparticles; Thi, thionine; Fc, ferrocene; ASDA, autocatalytic strand displacement amplification; AgNCs, silver nanoclusters; CuCo-CeO_2_, Cu/Co double-doped CeO_2_ nanospheres; TAPNR, target assisted polymerization nicking reaction; CHA, catalytic hairpin assembly; Exo-III, exonuclease III; MB, methylene blue; Exo I, exonuclease I; RuHex, hexaammineruthenium(III) chloride.

**Table 2:** Analytical performances of different electrochemical methods for miRNA-21 detection.

Signal Amplification	Linear Range	Detection Limit	Ref.
AuNPs-HRP	5 fM~0.5 μM	0.54 fM	[58]
DSN + AuNPs-HRP	0.1 fM~0.1 nM	43.3 aM	[59]
ALP + redox cycling	2 fM~1 nM	2 fM	[60]
ALP + redox cycling	0.5 fM~1 pM	0.2 fM	[61]
DSN + FeNxC network	0.5 fM~1 pM	0.2805 fM	[62]
CRISPR-Cas13a + CHA	10 fM~1 nM	2.6 fM	[63]
DSN + rGO	0.05 fM~5 fM	0.01 fM	[64]
T4 ligase + phi29	0.1 fM~100 pM	10.6 aM	[65]
EBFCs + CHA + HCR	0.5 fM~10 pM	0.15 fM	[66]
HCR + GOx	1 fM	1~500 fM	This work

Abbreviations: AuNPs, gold nanoparticles; HRP, horseradish peroxidase; DSN, duplex-specific nuclease; ALP, alkaline phosphatase; CRISPR-Cas13a, clustered regularly interspaced palindromic repeats/CRISPR-associated proteins system; CHA, catalytic hairpin assembly; rGO, reduced graphene oxide; phi29, phi 29 polymerase; EBFCs, enzymatic biofuel cells.

## Data Availability

Not applicable.

## References

[1] Dirks R.M., Pierce N.A. (2004). Triggered amplification by hybridization chain reaction. Proc. Natl. Acad. Sci. USA.

[2] Chai H., Cheng W., Jin D., Miao P. (2021). Recent progress in DNA hybridization chain reaction strategies for amplified biosensing. ACS Appl. Mater. Interfaces.

[3] Zhang C., Chen J., Sun R., Huang Z., Luo Z., Zhou C., Wu M., Duan Y., Li Y. (2020). The recent development of hybridization chain reaction strategies in biosensors. ACS Sens..

[4] Park C.R., Park S.J., Lee W.G., Hwang B.H. (2018). Biosensors using hybridization chain reaction—Design and signal amplification strategies of hybridization chain reaction. Biotech. Bioproc. Eng..

[5] Yang D., Tang Y., Miao P. (2017). Hybridization chain reaction directed DNA superstructures assembly for biosensing applications. TrAC-Trend. Anal. Chem..

[6] Liu N., Huang F., Lou X., Xia F. (2016). DNA hybridization chain reaction and DNA supersand wich self-assembly for ultrasensitive detection. Sci. China Chem..

[7] Augspurger E.E., Rana M., Yigit M.V. (2018). Chemical and biological sensing using hybridization chain reaction. ACS Sens..

[8] Feng Y., Xie H., Zhou B. (2022). Progress in hybridization chain reaction-based photoelectrochemical biosensors. Int. J. Electrochem. Sci..

[9] Sun T., Du J., Li Z., Zhao F. (2022). Recent Advancement in the development of hybridization chain reaction-based electrochemiluminescence biosensors. Int. J. Electrochem. Sci..

[10] Zhang Q. (2022). Application of hybridization chain reaction (HCR) in electrochemical analysis. Int. J. Electrochem. Sci..

[11] Singh A., Sharma A., Ahmed A., Sundramoorthy A.K., Furukawa H., Arya S., Khosla A. (2021). Recent advances in electrochemical biosensors: Applications, challenges, and future scope. Biosensors.

[12] Li H., Li X., Li S., Shang Z., Li H., Xia F. (2022). Electrochemical biosensors employing hybridization chain reaction: From structural design to applications. Adv. Mater. Interfaces.

[13] Qing M., Chen S.L., Sun Z., Fan Y., Luo H.Q., Li N.B. (2021). Universal and programmable rolling circle amplification-CRISPR/Cas12a-mediated immobilization-free electrochemical biosensor. Anal. Chem..

[14] Xie X., Wang Z., Zhou M., Xing Y., Chen Y., Huang J., Cai K.A., Zhang J. (2021). Redox host–guest nanosensors installed with DNA gatekeepers for immobilization-free and ratiometric electrochemical detection of miRNA. Small Methods.

[15] Guan Y., Wang F.P., Chen Z.X., Yang Y.H., Yang T., Hu R. (2023). Ratiometrically homogeneous electrochemical biosensor based on the signal amplified strategy of dual DNA nanomachines for microRNA analysis. Talanta.

[16] Yang L.M., Yin X.H., An B., Li F. (2021). Precise capture and direct quantification of tumor exosomes via highly efficient dual-aptamer recognition-assisted ratiometric immobilization-free electrochemical strategy. Anal. Chem..

[17] Li H., Qi H., Chang J., Gai P., Li F. (2022). Recent progress in homogeneous electrochemical sensors and their designs and applications. TrAC-Trend. Anal. Chem..

[18] Zhang F., Cai L., Zhou Y., Zhang X. (2016). Immobilization-free DNA-based homogeneous electrochemical biosensors. TrAC Trends Anal. Chem..

[19] Zhu L., Yu L., Yang X. (2021). Electrochemical-based DNA logic devices regulated by the diffusion and intercalation of electroactive dyes. ACS Appl. Mater. Interfaces.

[20] Hou T., Li W., Liu X., Li F. (2015). Label-free and enzyme-free homogeneous electrochemical biosensing strategy based on hybridization chain reaction: A facile, sensitive, and highly specific microRNA assay. Anal. Chem..

[21] Wang H., Yang C., Tang H., Li Y. (2021). Stochastic collision electrochemistry from single G-quadruplex/hemin: Electrochemical amplification and microRNA sensing. Anal. Chem..

[22] Liao X., Zhang C., Machuki J.O., Wen X., Chen D., Tang Q., Gao F. (2021). Proximity hybridization triggered hybridization chain reaction for label-free electrochemical homogeneous aptasensors. Talanta.

[23] Xuan F., Luo X., Hsing I.-M. (2012). Ultrasensitive solution-phase electrochemical molecular beacon-based DNA detection with signal amplification by exonuclease III assisted target recycling. Anal. Chem..

[24] Liu S., Lin Y., Wang L., Liu T., Cheng C., Wei W., Tang B. (2014). Exonuclease III-aided autocatalytic DNA biosensing platform for immobilization-free and ultrasensitive electrochemical detection of nucleic acid and protein. Anal. Chem..

[25] Lu L., Su H., Li F. (2017). Ultrasensitive homogeneous electrochemical detection of transcription factor by coupled isothermal cleavage reaction and cycling amplification based on exonuclease III. Anal. Chem..

[26] Chen J.-Y., Liu Z.-J., Wang X.-W., Ye C.-L., Zheng Y.-J., Peng H.-P., Zhong G.-X., Liu A.-L., Chen W., Lin X.-H. (2019). Ultrasensitive electrochemical biosensor developed by probe lengthening for detection of genomic DNA in human serum. Anal. Chem..

[27] Chang J.F., Wang X., Wang J., Li H.Y., Li F. (2019). Nucleic acid functionalized MOFs-based homogeneous electrochemical biosensor for simultaneous detection of multiple tumor biomarkers. Anal. Chem..

[28] Chang J.F., Lv W.X., Li Q., Li H.Y., Li F. (2020). One-step synthesis of methylene blue-encapsulated ZIF for dual-signal fluorescent and homogeneous electrochemical biosensing. Anal. Chem..

[29] Liu L., Chang Y., Ji X., Chen J., Zhang M., Yang S. (2023). Surface-tethered electrochemical biosensor for telomerase detection by integration of homogeneous extension and hybridization reactions. Talanta.

[30] Xia N., Sun T., Liu L., Tian L., Sun Z. (2022). Heterogeneous sensing of post-translational modification enzymes by integrating the advantage of homogeneous analysis. Talanta.

[31] Xia N., Sun Z., Ding F., Wang Y., Sun W., Liu L. (2021). Protease biosensor by conversion of a homogeneous assay into a surface-tethered electrochemical analysis based on streptavidin-biotin interactions. ACS Sens..

[32] Liu L., Deng D., Wu D., Hou W., Wang L., Li N., Sun Z. (2021). Duplex-specific nuclease-based electrochemical biosensor for the detection of microRNAs by conversion of homogeneous assay into surface-tethered electrochemical analysis. Anal. Chim. Acta.

[33] Cai A., Yang L., Kang X., Liu J., Wang F., Ji H., Wang Q., Wu M., Li G., Zhou X. (2022). Target recognition- and HCR amplification-induced in situ electrochemical signal probe synthesis strategy for trace ctDNA analysis. Biosensors.

[34] Grabowska I., Hepel M., Kurzątkowska-Adaszyńs K. (2022). Advances in design strategies of multiplex electrochemical aptasensors. Sensors.

[35] Khan S., Burciu B., Filipe C.D.M., Li Y., Dellinger K., Didar T.F. (2021). DNAzyme-based biosensors: Immobilization strategies, applications, and future prospective. ACS Nano.

[36] Chang Y., Ma X., Sun T., Liu L., Hao Y. (2021). Electrochemical detection of kinase by converting homogeneous analysis into heterogeneous assay through avidin-biotin interaction. Talanta.

[37] Povedano E., Montiel V.R.V., Gamella M., Pedrero M., Barderas R., Pelaez-Garcia A., Mendiola M., Hardisson D., Feliu J., Yanez-Sedeno P. (2020). Amperometric bioplatforms to detect regional DNA methylation with single-base sensitivity. Anal. Chem..

[38] Yu S., Chen S., Dang Y., Zhou Y., Zhu J.-J. (2022). An ultrasensitive electrochemical biosensor integrated by nicking endonuclease-assisted primer exchange reaction cascade amplification and DNA nanosphere-mediated electrochemical signal-enhanced system for microRNA detection. Anal. Chem..

[39] Li Y., Ling L. (2015). Aptamer-based fluorescent solid-phase thrombin assay using a silver-coated glass substrate and signal amplification by glucose oxidase. Microchim. Acta.

[40] Liu X., Shuai H.-L., Liu Y.-J., Huang K.-J. (2016). An electrochemical biosensor for DNA detection based on tungsten disulfide/multi-walled carbon nanotube composites and hybridization chain reaction amplification. Sens. Actuat. B Chem..

[41] Ge Z., Lin M., Wang P., Pei H., Yan J., Shi J., Huang Q., He D., Fan C., Zuo X. (2014). Hybridization chain reaction amplification of microRNA detection with a tetrahedral DNA nanostructure-based electrochemical biosensor. Anal. Chem..

[42] Wan Y., Wang H., Ji J., Kang K., Yang M., Huang Y., Su Y., Ma K., Zhu L., Deng S. (2020). Zippering DNA tetrahedral hyperlink for ultrasensitive electrochemical microRNA detection. Anal. Chem..

[43] Zhai F., Yu Q., Zhou H., Liu J., Yang W., You J. (2019). Electrochemical selective detection of carnitine enantiomers coupling copper ion dependent DNAzyme with DNA assistant hybridization chain reaction. J. Electroanal. Chem..

[44] Wang C., Zhou H., Zhu W., Li H., Jiang J., Shen G., Yu R. (2013). Ultrasensitive electrochemical DNA detection based on dual amplification of circular strand-displacement polymerase reaction and hybridization chain reaction. Biosens. Bioelectron..

[45] Luo S., Zhang Y., Huang G., Situ B., Ye X., Tao M., Huang Y., Li B., Jiang X., Wang Q. (2021). An enzyme-free amplification strategy for sensitive assay of circulating tumor DNA based on wheel-like catalytic hairpin assembly and frame hybridization chain reaction. Sens. Actuat. B Chem..

[46] Miao P., Tang Y., Yin J. (2015). MicroRNA detection based on analyte triggered nanoparticle localization on a tetrahedral DNA modified electrode followed by hybridization chain reaction dual amplification. Chem. Commun..

[47] Yuan Y.H., Wu Y.D., Chi B.Z., Wen S.H., Liang R.P., Qiu J.D. (2017). Simultaneously electrochemical detection of microRNAs based on multifunctional magnetic nanoparticles probe coupling with hybridization chain reaction. Biosens. Bioelectron..

[48] Chen Z., Liu Y., Xin C., Zhao J., Liu S. (2018). A cascade autocatalytic strand displacement amplification and hybridization chain reaction event for label-free and ultrasensitive electrochemical nucleic acid biosensing. Biosens. Bioelectron..

[49] Yang C., Shi K., Dou B., Xiang Y., Chai Y., Yuan R. (2015). In situ DNA-templated synthesis of silver nanoclusters for ultrasensitive and label-free electrochemical detection of microRNA. ACS Appl. Mater. Interfaces.

[50] Zhou C., Cui K., Liu Y., Li L., Zhang L., Xu M., Ge S., Wang Y., Yu J. (2020). Ultrasensitive lab-on-paper device via Cu/Co double-doped CeO_2_ nanospheres as signal amplifiers for electrochemical/visual sensing of miRNA-155. Sens. Actuat. B Chem..

[51] Ren W., Gao Z.F., Li N.B., Luo H.Q. (2015). Ultrasensitive and selective signal-on electrochemical DNA detection via exonuclease III catalysis and hybridization chain reaction amplification. Biosens. Bioelectron..

[52] Cheng H., Li W., Duan S., Peng J., Liu J., Ma W., Wang H., He X., Wang K. (2019). Mesoporous silica containers and programmed catalytic hairpin assembly/hybridization chain reaction based electrochemical sensing platform for microRNA ultrasensitive detection with low background. Anal. Chem..

[53] Guo Q., Yu Y., Zhang H., Cai C., Shen Q. (2020). Electrochemical sensing of exosomal microRNA based on hybridization chain reaction signal amplification with reduced false-positive signals. Anal. Chem..

[54] Negahdary M., Angnes L. (2022). Application of electrochemical biosensors for the detection of microRNAs (miRNAs) related to cancer. Coord. Chem. Rev..

[55] Aziz N.B., Mahmudunnabi R.G.G., Umer M., Sharma S., Rashid M.A., Alhamhoom Y., Shim Y.B., Salomon C., Shiddiky M.J.A. (2020). MicroRNAs in ovarian cancer and recent advances in the development of microRNA-based biosensors. Analyst.

[56] Gillespie P., Ladame S., O’Hare D. (2019). Molecular methods in electrochemical microRNA detection. Analyst.

[57] Liang T., Qin X., Xiang Y., Tang Y., Yang F. (2022). Advances in nucleic acids-scaffolded electrical sensing of extracellular vesicle biomarkers. TrAC-Trend. Anal. Chem..

[58] Dong J., Yang H., Zhao J., Wen L., He C., Hu Z., Li J., Huo D., Hou C. (2022). Sandwich-type microRNA biosensor based on graphene oxide incorporated 3D-flower-like MoS_2_ and AuNPs coupling with HRP enzyme signal amplification. Microchim. Acta.

[59] Zhang H., Fan M., Jiang J., Shen Q., Cai C., Shen J. (2019). Sensitive electrochemical biosensor for MicroRNAs based on duplexspecific nuclease-assisted target recycling followed with gold nanoparticles and enzymatic signal amplification. Anal. Chim. Acta..

[60] Fang C.S., Kim K.-s., Yu B., Jon S., Kim M.-S., Yang H. (2017). Ultrasensitive electrochemical detection of miRNA-21 using a zinc finger protein specific to DNA–RNA hybrids. Anal. Chem..

[61] Xia N., Zhang Y., Wei X., Huang Y., Liu L. (2015). An electrochemical microRNAs biosensor with the signal amplification of alkaline phosphatase and electrochemical–chemical–chemical redox cycling. Anal. Chim. Acta.

[62] Ning Y., Zhang C., Wang C., Zhou C., Gong N., Wang Q., Zhu Y. (2022). DNA self-assembled FeNxC nanocatalytic network for ultrasensitive electrochemical detection of microRNA. Anal. Chim. Acta.

[63] Cui Y., Fan S., Yuan Z., Song M., Hu J., Qian D., Zhen D., Li J., Zhu B. (2021). Ultrasensitive electrochemical assay for microRNA-21 based on CRISPR/Cas13a-assisted catalytic hairpin assembly. Talanta.

[64] Xiao Q., Li J., Jin X., Liu Y., Huang S. (2019). Ultrasensitive electrochemical microRNA-21 biosensor coupling with carboxylate-reduced graphene oxide-based signal-enhancing and duplexspecific nuclease-assisted target recycling. Sens. Actuat. B Chem..

[65] Zhu L., Zhang X., Yuan R., Chai Y. (2023). Y-shaped walker with abundant recognition domians mediated ultrasensitive electrochemical biosensor for miRNA-21 detection. Sens. Actuat. B Chem..

[66] Wang F., Cai R., Tan W. (2023). Self-powered biosensor for a highly efficient and ultrasensitive dual-biomarker assay. Anal. Chem..

[67] Zhang X., Yang Z., Chang Y., Qing M., Yuan R., Chai Y. (2018). A novel 2D DNA nanoprobe mediated enzyme-free target recycling amplification for ultrasensitive electrochemical detection of microRNA. Anal. Chem..

